# Risk Factors for Hospital Admission with RSV Bronchiolitis in England: A Population-Based Birth Cohort Study

**DOI:** 10.1371/journal.pone.0089186

**Published:** 2014-02-26

**Authors:** Joanna Murray, Alex Bottle, Mike Sharland, Neena Modi, Paul Aylin, Azeem Majeed, Sonia Saxena, on behalf of the Medicines for Neonates Investigator Group

**Affiliations:** 1 Department of Primary Care and Public Health, Imperial College London, London, United Kingdom; 2 Paediatric Infectious Diseases Unit, St. George's Hospital NHS Trust, London, United Kingdom; 3 Section of Neonatal Medicine, Department of Medicine, Imperial College London, London, United Kingdom; Kliniken der Stadt Köln gGmbH, Germany

## Abstract

**Objective:**

To examine the timing and duration of RSV bronchiolitis hospital admission among term and preterm infants in England and to identify risk factors for bronchiolitis admission.

**Design:**

A population-based birth cohort with follow-up to age 1 year, using the Hospital Episode Statistics database.

**Setting:**

71 hospitals across England.

**Participants:**

We identified 296618 individual birth records from 2007/08 and linked to subsequent hospital admission records during the first year of life.

**Results:**

In our cohort there were 7189 hospital admissions with a diagnosis of bronchiolitis, 24.2 admissions per 1000 infants under 1 year (95%CI 23.7–24.8), of which 15% (1050/7189) were born preterm (47.3 bronchiolitis admissions per 1000 preterm infants (95% CI 44.4–50.2)). The peak age group for bronchiolitis admissions was infants aged 1 month and the median was age 120 days (IQR = 61–209 days). The median length of stay was 1 day (IQR = 0–3). The relative risk (RR) of a bronchiolitis admission was higher among infants with known risk factors for severe RSV infection, including those born preterm (RR = 1.9, 95% CI 1.8–2.0) compared with infants born at term. Other conditions also significantly increased risk of bronchiolitis admission, including Down's syndrome (RR = 2.5, 95% CI 1.7–3.7) and cerebral palsy (RR = 2.4, 95% CI 1.5–4.0).

**Conclusions:**

Most (85%) of the infants who are admitted to hospital with bronchiolitis in England are born at term, with no known predisposing risk factors for severe RSV infection, although risk of admission is higher in known risk groups. The early age of bronchiolitis admissions has important implications for the potential impact and timing of future active and passive immunisations. More research is needed to explain why babies born with Down's syndrome and cerebral palsy are also at higher risk of hospital admission with RSV bronchiolitis.

## Introduction

Acute bronchiolitis is the most common lower respiratory tract infection (LRTI) in infants affecting around 10% of children under 1 year [1]–[5]. It is usually a mild, self-limiting illness but in some infants may be more severe, requiring hospital admission. Bronchiolitis is typically caused by respiratory syncytial virus (RSV) in around 75% of cases [6], with peak incidence during the winter months. Bronchiolitis admission rates in infants under 1 year have previously been estimated from small regional studies to be 30·8 per 1000 in the United Kingdom (UK) [3] and 31·2 per 1000 in the United States (US) [7]. To date, no national studies have reported the disease burden at a population level in the UK.

Children known to be at high risk of developing severe RSV infection include those with chronic lung disease, congenital heart disease, immunodeficiency, low birth weight and those born preterm (approximately 8% of newborns in the UK) [8]–[11]. Other factors associated with RSV infection include winter births, poverty, malnutrition, tobacco smoke exposure, lack of breast-feeding and multiple gestation [12]. Evidence from the US suggests most children admitted to hospital with RSV infection are healthy, with no risk factors for severe RSV infection [9].

Since there is no effective drug treatment, the mainstay of management for infants with RSV bronchiolitis is supportive care and a small proportion of more severely affected infants are treated in intensive care [1], [13]–[15]. Prophylactic passive immunotherapy with Palivizumab (Synagis®, MedImmune), a monoclonal antibody against RSV infection, is licensed for use in some countries for those most at high risk of RSV infection, but is very expensive (the estimated cost for a single dose of Palivizumab for an infant aged 6 months, weighing 7·5 kg, is £1023·11 [16] so the total cost for 5 doses over the winter season is just over £5000 per patient). The Joint Committee for Vaccination and Immunisation (JCVI) in the UK states Palivizumab use is only cost-effective for a small group of infants at most risk of severe disease [16]–[18], but there remains a poor evidence base of prognostic factors for hospital admission due to RSV infection [18]. An active immunisation against RSV is currently in phase III trials, also aimed primarily at high-risk groups. However, the difficulty of eliciting immunity in such young infants remains a challenge. The aim of this study was to examine which infants are most at risk of an RSV bronchiolitis admission in England in term, preterm and high-risk infants and to determine the age at and duration of an RSV bronchiolitis admission.

## Methods

### Data

Hospital Episodes Statistics (HES) is a national administrative database, electronically recording all admissions to NHS hospitals in England since 1989 (www.hesonline.nhs.uk) [19] and has been used extensively for research and measuring quality of healthcare delivery [20], [21]. Annual reports from the Office for National Statistics suggest that around 97% of all live births in England occur in NHS hospitals. Coders enter information about clinical diagnoses undertaken using the International Classification of Diseases 10^th^ revision (ICD-10). Maternity data in HES consists of delivery records (for mothers) and birth records (for babies). These contain additional data fields known as the “baby tail”, which include information such as birth weight and gestational age [22]–[24].

### Study cohort

We created a birth cohort for all infants born in English NHS hospitals and discharged during a twelve month period (from 1^st^ April 2007 to 31^st^ March 2008). We included only records from live births and excluded infants born in hospitals (85/156) with poor recording (<90% complete) of key indicators (birth weight and gestational age), to enable us to group infants into term and preterm categories [24]. Sensitivity analyses of number of maternity beds, annual number of births, geographic location and infant death rate were conducted to compare hospitals in the study with sites excluded because of poor recording [24]. We linked birth records to subsequent hospital admission records up to a child's first birthday, using a unique personal identifier (HES ID). Linkage to Office of National Statistics (ONS) mortality records allowed us to identify any deaths among the birth cohort during their first year of life, including out-of-hospital deaths.

### Outcome and exposure measures

Diagnostic information recorded in individual birth records and any subsequent hospital admission records from the study year was used to group infants into categories of risk factors for severe RSV infection (Appendix S1) using ICD-10 codes or larger subgroups using the Agency for Health Research and Quality's Clinical Classification System (CCS) [25]. Infants were considered preterm if their gestational age at birth was less than 37 weeks (Appendix S1), in accordance with the World Health Organisation (WHO) definition of premature birth [26]. Infants missing gestational age were assumed to be born at full term.

We identified infants admitted as an emergency with a primary diagnosis of acute bronchiolitis using the ‘J21’ ICD-10 codes (‘J210’ - acute bronchiolitis due to respiratory syncytial virus, ‘J218’ - acute bronchiolitis due to other specified organisms and ‘J219’ - acute bronchiolitis, unspecified). Most bronchiolitis admissions were coded with unspecific aetiology and approximately a third were coded as being due to RSV. We grouped all bronchiolitis codes into a single “RSV bronchiolitis” category as RSV is the commonest cause of bronchiolitis, but specific microbiological diagnoses are poorly coded in HES. We excluded bronchiolitis admissions in children over 1 year because of the uncertain nature of this clinical diagnosis in older infants. We defined infants as being under 1 year of age. We examined age at bronchiolitis admission and calculated the median length of stay (LOS) for bronchiolitis admissions to provide a proxy measure of severity of illness.

### Statistical analyses

We calculated the absolute risk of a bronchiolitis admission among infants with and without risk factors for severe RSV infection, including preterm infants and infants with a clinical diagnosis of cystic fibrosis, congenital heart disease, nervous system congenital anomalies, other congenital anomalies, immunodeficiency, cerebral palsy or Down's syndrome (case definitions and codes in Appendix S1). Infants without a particular risk factor condition were considered “healthy”. Associated 95% confidence intervals (CI) were calculated using Poisson approximation. We calculated the relative risk (RR) of a bronchiolitis admission, with associated 95% CI for infants in each individual risk group, by comparing with the baseline group of infants without the particular risk factor. Infants may belong to more than 1 of these risk groups, so we controlled for this potential confounding using Poisson regression models to calculate the adjusted RR of bronchiolitis admission for infants in each risk group. To test for significant differences between proportions we used Chi Squared tests and Mann-Whitney tests to compare median values for non-normal data. Data were analysed using the SAS 9·2 software package (SAS Institute, Cary, N.C., USA).

### Research Ethics approval

We have permission from the National Information Governance Board (NIGB) under Section 251 of the NHS Act 2006 (formerly Section 60 approval from the Patient Information Advisory Group) to hold confidential data. We have approval to use them for research and measuring quality of delivery of healthcare, from the South East Ethics Research Committee.

## Results

The birth cohort included 296618 infants, from 71 NHS hospitals in England. 410 infants in the cohort died during the study year. 51% (151897/296618) of the cohort were boys, 1% (2891) were multiple births and 7·5% (22215) were born preterm before 37 weeks gestation (Table S2, Appendix S2).

**Figure 1:**
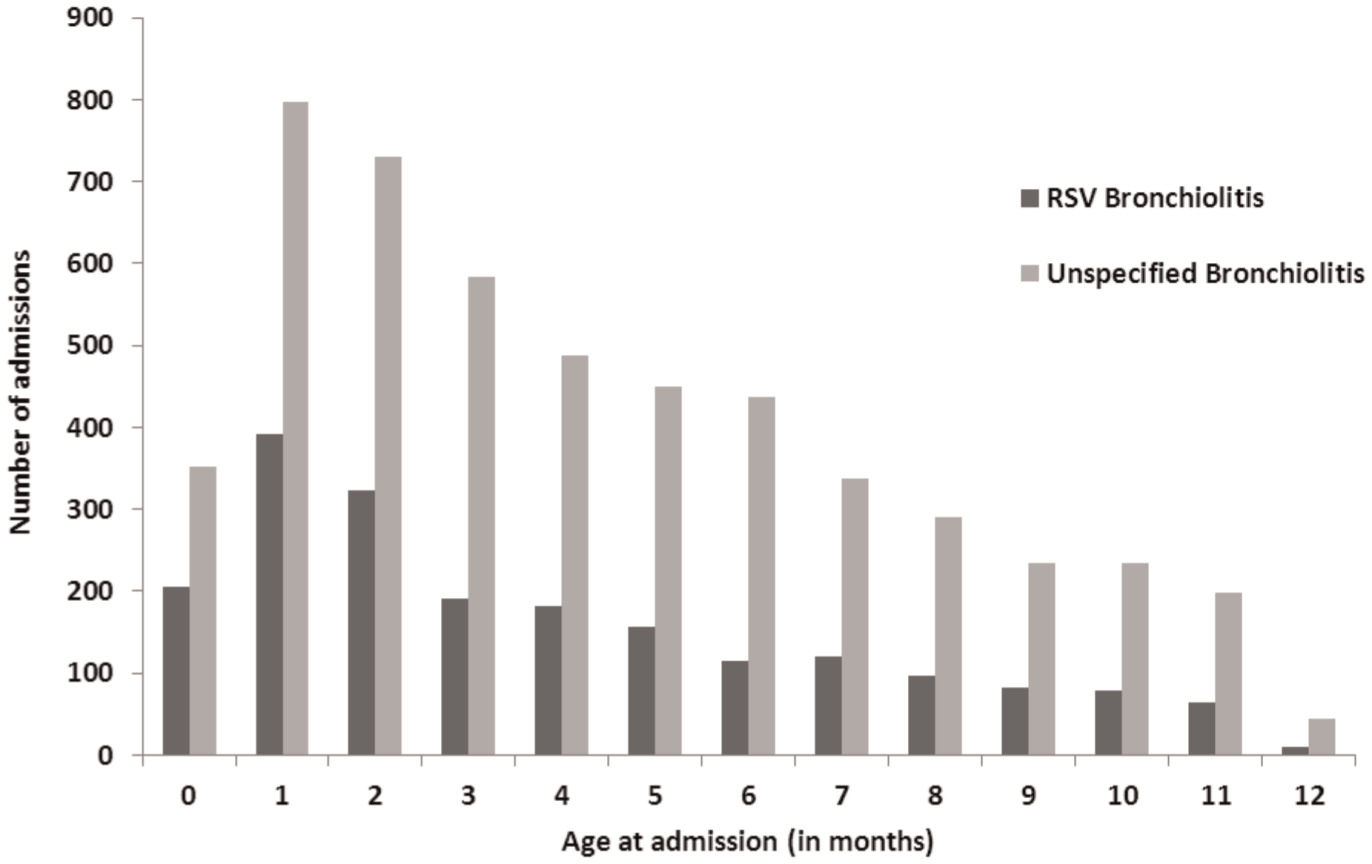
Age of bronchiolitis admission in months, for admissions coded as RSV or unspecified bronchiolitis.

Among our birth cohort there were 7189 admissions to hospital with a primary diagnosis of bronchiolitis during their first year of life, 24·2 admissions per 1000 infants (95% CI 23·7 to 24·8). Only 2015 (28·0%) of these were specifically coded as being due to RSV, the remainder were unspecified. In total, 1529 (21·3%) infants admitted with bronchiolitis, had more than one bronchiolitis admission during their first year of life. The modal age of bronchiolitis admission was 1 month (Figure 1) and the median age was 120 days (Inter-quartile range (IQR) = 61 to 209). The median length of hospital stay was 1 day (IQR = 0 to 3 days).

Among infants admitted with bronchiolitis, 15% (1050/7189) were born preterm. Admission rates were higher among infants born preterm (47·3 per 1000 infants (95%CI 44·4–50·2)) compared with those born at term (22·4 per 1000 infants (95%CI 21·8–22·9)) (Table 1). The median length of stay for a bronchiolitis admission among infants born preterm was 1 day (IQR = 0 to 3) and among those born at term it was also 1 day (IQR = 0 to 3). The median age of the bronchiolitis admission among infants born preterm was 136 days (IQR = 71 to 221) compared with 118 days (IQR = 59 to 207) among infants born at term (p<0·001).

**Table 1:** Rates and relative risk of a bronchiolitis hospital admission among infants in different risk groups.

Risk Group†	Number of bronchiolitis admissions (% of infants in risk group)	Total number of infants in risk group (% of whole birth cohort)	Median length of stay in days (IQR)	Rate of bronchiolitis admission per 1000 infants under 1 year (95% CIs)	RR† (95% CIs)	Adjusted RR (95%CI)
**Born at term**	6139 (2·2)	274403 (92·5)	1 (0 to 3)	22·4 (21·8 to 22·9)	–	–
**Premature birth**	1050 (4·7)	22215 (7·5)	1 (0 to 3)	47·3 (44·4 to 50·2)	2·11 (1·98 to 2·26)	1.89 (1·77 to 2·02)
**Cystic fibrosis**	11 (6·4)	171 (0·1)	2 (0 to 14)	64·3 (32·1 to 115·1)	2·66 (1·33 to 4·76)	2·45 (1·36 to 4·43)
**Congenital heart Disease**	272 (12·1)	2239 (0·8)	2 (0 to 5)	121·5 (107·5 to 136·8)	5·17 (4·56 to 5·84)	3·35 (2·92 to 3·84)
**Chronic lung disease**	282 (5·6)	5016 (1·7)	2 (0 to 4)	56·2 (49·9 to 63·2)	2·37 (2·10 to 2·67)	1·61 (1·42 to 1·82)
**Immunodeficiency**	7 (11·7)	60 (0·0)	8 (1 to 58)	116·7 (46·9 to 240·4)	4·82 (1·94 to 9·93)	1·69 (0·80 to 3·58)
**Nervous system congenital anomalies**	42 (8·6)	489 (0·2)	2 (1 to 4)	85·9 (61·9 to 116·1)	3·56 (2·56 to 4·82)	1·73 (1·26 to 2·36)
**Down's syndrome**	28 (15·4)	182 (0·1)	3 (0 to 9)	153·9 (102·2 to 222·4)	6·37 (4·23 to 9·21)	2·53 (1·72 to 3·72)
**Cerebral Palsy**	16 (10·7)	149 (0·1)	3 (1 to 5)	107·4 (61·4 to 174·4)	4·44 (2·54 to 7·21)	2·43 (1·48 to 3·99)

† Relative risk compared with infants without risk factor of interest.

**Figure 2:**
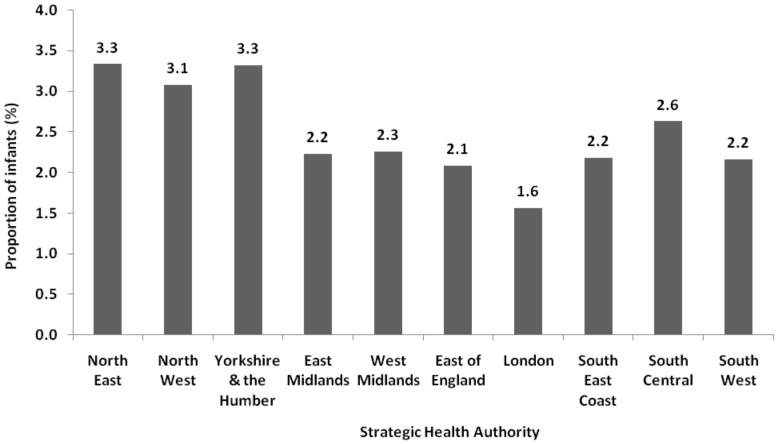
Proportion of bronchiolitis hospital admissions among birth cohort, by Strategic Health Authority (SHA) of admission.

Among infants admitted with bronchiolitis, 1722 (24·0%) had one or more known risk factors for severe RSV infection. The adjusted RR of a bronchiolitis admission was high among infants with known risk factors for severe RSV disease, including those born preterm (RR 1·9 (95%CI 1·8–2·0)) or with congenital heart conditions (RR 3·4 (95%CI 2·9–3·8)) or chronic lung disease (RR 1·6 (95%CI 1·4–1·8)), compared with healthy infants without the risk factor (Table 1). Other conditions also significantly increased an infant's risk of bronchiolitis admission, including cystic fibrosis (RR = 2·5 (95% CI 1·4 to 4·4)), Down's syndrome (RR 2·5 (95%CI 1·7–3·7)), cerebral palsy (RR 2·4 (95%CI 1·5–4·0)) and other nervous system congenital abnormalities (RR = 1·7 (95% CI 1·3 to 2·4)). A higher proportion of infants born in September (3·9%) or October (3·8%) were admitted with bronchiolitis than among infants born in any other month (p<0·001). Bronchiolitis admission rates among infants born in the North East (3·3%) and Yorkshire and the Humber (3·3%) SHAs, were higher (p<0·001) compared with in infants born in London (1·6%) (Figure 2).

## Discussion

### Main findings

Most (85%) of the infants who are admitted to hospital with bronchiolitis in England are born at term, with no known predisposing risk factors for severe RSV infection, although risk of admission is higher in known risk groups. Babies born with Down's syndrome, cerebral palsy and cystic fibrosis are also at higher risk of hospital admission with RSV bronchiolitis. The early age of bronchiolitis admissions has important implications for the potential impact and timing of future active and passive immunisations.

### Strengths and weaknesses

This is among the first studies reporting the population burden of bronchiolitis across NHS hospitals in England. The main strength of our cohort is that use of HES birth records provides a large, nationally representative sample of births across England during this time, when compared with ONS live births data [27]. We assumed infants with unknown gestational age and birth weight were not born preterm, as infants in this unknown group had similarly short length of stay at birth to infants born at term. Despite incomplete recording of gestational age in some birth records, 7·5% of our cohort were born at <37 weeks gestation which is consistent with ONS data reporting that around 7·7% of live births annually are delivered preterm [28]. This suggests under-reporting of gestational age in HES birth records appears to be mostly among term babies. Similarly, rates of congenital heart disease, chronic lung disease and other risk factors for severe RSV disease compare well with published UK population data [3], [29], [30].

However, there are a number of important limitations to our study. Our case definition for bronchiolitis and co-morbidity is dependent on the accuracy of clinical coding and recording in diagnosis fields in HES records, although improvements in the quality of HES coding have been demonstrated in recent years [31]. We combined RSV and unspecified bronchiolitis, presenting data on *all* bronchiolitis admissions. Only 28% of the bronchiolitis admissions were coded as being due to RSV, the remainder having unspecific bronchiolitis codes. No linked microbiological data to confirm the presence of RSV was available. A previous study in the UK has estimated that around 75% of unspecified bronchiolitis admissions were RSV related [32]. Globally it is estimated that only between 4% and 28% of children admitted with LRTI are tested for RSV [4].

A limitation of the cohort study design is attrition, as it was not possible to identify bronchiolitis admissions occurring outside NHS hospitals in England, or to identify individuals who migrated elsewhere, though we estimate this number should be low with only one year of follow-up. Using linked ONS mortality data it was possible to identify the small number of individuals in the cohort who died during the study period. The number of infant deaths directly attributable to RSV infection is reported to be very low [33]. There is potentially some inherent selection bias in our study, through the inclusion of only hospitals with good completeness of recording of birth record fields, but sensitivity analyses showed few differences between included and excluded sites in terms of supply factors (such as number of maternity beds and number of deliveries). No data were available to identify any individuals who had received Palivizumab prophylaxis against RSV infection. There are very strict criteria for Palivizumab use in the UK due to its significant cost, which means that very few babies in the cohort are likely to have received it.

### Findings in relation to other studies

We found no comparable national studies to compare our estimates of bronchiolitis hospital admission rates in the UK but one previous study from Shropshire reported a bronchiolitis hospital admission rate of 30·8 per 1000 infants in this region of England [3], compared with our estimate of 24·2 admissions per 1000 infants. Our estimate may be slightly lower because we applied a narrow case definition including only infants admitted with a primary diagnosis of bronchiolitis (ICD-10 ‘J21’codes). This is likely to underestimate the total burden of RSV illness in infancy, as we have excluded pneumonias due to RSV and non-specific, symptomatic clinical codes from our bronchiolitis case definition, though some of these admissions may in reality have been related to RSV infection. A study from the United States has reported a bronchiolitis admission rate of 31·2 per 1000 infants under 1 year [7], but a more recent study in Texas reported higher bronchiolitis admission rates of 5·5% among children under 2 years [34]. These higher incidence estimates are likely to be due to the wider inclusion of children aged 12 to 24 months in their study, coupled with variation in the clinical definition of bronchiolitis used in the US compared with that used in UK [13].

21% of infants admitted with bronchiolitis, had more than one bronchiolitis admission during their first year of life. Previous infection with RSV only confers partial immunity, so it is possible for individuals to be re-infected with the same or different RSV strains over the course of one season. Re-admissions for bronchiolitis among older infants may in reality be incorrectly diagnosed episodes of post-bronchiolitic wheezing. The modal age of bronchiolitis admission was 1 month and the median was 4 months, confirming findings reported elsewhere which show increased risk of RSV admission with younger age [13], [35]–[37]. It is unclear why RSV may be affecting babies at a younger age than has previously been reported. It could be associated with changes to the virus itself or maternal immunological priming, but further investigation into what may be driving this trend is needed. The median age of bronchiolitis admission was 4 months which is consistent with other studies where RSV hospital admissions are typically reported during the first 3 to 6 months of life [8], [38]. However, there is some debate regarding the most common age of RSV admissions [37]. A population-based cohort in Denmark found the median age of admission for RSV infections was 6 months [39]. Similarly, a recent prognostic birth cohort study reported that the median age at time of RSV LRTI was 6 months [40]. Unplanned short stay hospital admissions in this age group have been increasing [20]. In 2006 the unplanned admission rate among children less than 1 year in England was 181 per 1000 child years [20]. Our findings suggest that approximately 1 in 6 of these unplanned admissions was for bronchiolitis.

We found the majority of infants admitted with bronchiolitis were not in high-risk groups, with only 24% (1722/7189) having one or more recognised risk factors for severe RSV infection. In a smaller US study, 34% of RSV-infected patients (189/564) had at least one high-risk condition for RSV infection [9]. Preterm infants accounted for only 15% of bronchiolitis admissions in our study. Infants with chronic lung disease or congenital heart disease each accounted for around 4% of bronchiolitis admissions. This is consistent with findings reported elsewhere, suggesting that the majority of infants with severe RSV infection do not have any underlying risk factors [9]. Low rates of bronchiolitis admissions in some high-risk groups may also be driven by Palivizumab use, however no national prescribing data for Palivizumab is available for us to estimate the number of admissions its use may have prevented. The median age of bronchiolitis admission was only 18 days later in preterm infants (136 days) compared with those born at term (118 days), so onset of RSV disease appears to occur at a similar corrected age. Median LOS for bronchiolitis admissions was calculated as a proxy marker of severity of illness and was found to be similarly short (1 day) among both term and preterm born infants.

Geographical variation in bronchiolitis admission rates may be a proxy for other risk factors for hospital admission with severe RSV infection, such as maternal smoking or deprivation. We think this finding is most likely driven by variation in clinical practice, with different thresholds for admission in different regions as there are no national evidence based guidelines giving criteria for admission. Indeed, widespread variation in hospital management of bronchiolitis has previously been reported [13].

### Implications and future research

Our study has highlighted that the burden of RSV bronchiolitis hospital admissions among infants in England predominantly affects those born at term, without any risk factors for severe RSV infection and the age at admission appears to be significantly lower now than previously reported. Since the majority of infants hospitalised with bronchiolitis are below 6 months of age, this will limit the impact of any future vaccine which is unlikely to be capable of eliciting immunity in such young infants. This has important implications for future vaccination strategies, suggesting that the cost benefit of both active and passive vaccines needs detailed evaluation. We found that the median length of stay for bronchiolitis admissions was only 1 day. Improved management of symptoms in the community, through both primary care and A & E services may reduce the need for admission still further. Further studies examining the clinical burden of bronchiolitis in the community are needed and could confirm the clinical and health service burden to be greater still [13], [16], [18]. Improved management of RSV is achievable through the use of improved, evidence-based guidelines [1]. The regional variation in bronchiolitis admission rates we have presented suggests admission criteria vary widely and there is a need for clearer guidance about which infants to admit to hospital in the UK.

Our findings suggest that in addition to the recognised risk factors for RSV bronchiolitis hospital admission, namely congenital heart disease, chronic lung disease and preterm birth, several other clinical conditions may also increase risk. The factors we have identified are cerebral palsy, cystic fibrosis and Down's syndrome. We suggest further investigation is warranted, into whether infants with these conditions would also benefit from Palivizumab immunoprophylaxis.

## Conclusion

Bronchiolitis is an important cause of hospital admissions among infants in the UK. Several clinical subgroups are at increased risk of hospital admission, but the majority of bronchiolitis cases in England are among infants born at term, with no known risk factors for severe RSV infection. The early age of bronchiolitis admissions has important implications for the potential impact of future active and passive immunisations. More research is needed to explain why babies born with Down's syndrome and cerebral palsy are also at higher risk of hospital admission with RSV bronchiolitis.

### What's known on this subject

Well known predisposing risk factors for RSV bronchiolitis include preterm birth, chronic lung disease, congenital heart disease and immune deficiency. Passive immunotherapy is indicated for those at highest risk of severe infection.

### What this study adds

Most infants admitted to hospital with bronchiolitis are born at term and have no risk factors for severe RSV infection. There is a peak in bronchiolitis admissions at the age of 1 month.

### Research Ethics approval

We have permission from the NIGB under Section 251 of the NHS Act 2006 (formerly Section 60 approval from the Patient Information Advisory Group) to hold confidential data. We have approval to use them for research and measuring quality of delivery of healthcare, from the South East Ethics Research Committee.

## Supporting Information

Appendix S1 **Identifying at-risk groups (ICD-10 code lists).**
(DOCX)

Appendix S2 **Table S1: Characteristics of birth cohort and infants hospitalised with bronchiolitis.**
(DOCX)
