# Supplementary material for: Risk Factors for Hospital Admission with RSV Bronchiolitis in England: A Population-Based Birth Cohort Study
Source: PLoS One. 2014 Feb 26;9(2):e89186. doi: 10.1371/journal.pone.0089186 (PMC3935842; doi:10.1371/journal.pone.0089186)
Supplement: Appendix S1 — Identifying at-risk groups (ICD-10 code lists). (DOCX) [file pone.0089186.s001.docx]

# Appendix S1

## Identifying at-risk groups (ICD-10 code lists)

**Prematurity**

Babies were categorised as born prematurely if their gestational age at birth was <37 weeks.^26^

If a birth record had no gestational age recorded, i.e. premature status was unknown, then they were assumed to be not premature (justified on the basis that infants in the unknown group had similarly low ICU admission rates and short length of stay at birth, to infants in the group known to be born at term.)

**Immunodeficiency:** CCS group 57 – Immunity disorders (This includes ICD-10 codes D80, D81, D82, D83, D84 and D89 which includes diagnoses such as hypogammaglobulinemia and severe combined immunodeficiency.)

**Cystic fibrosis:** CCS group 56 – Cystic fibrosis (This includes ICD-10 codes under E84.)

**Chronic lung disease:** ICD-10 codes P27 – Chronic respiratory disease originating in the perinatal period and P28 – Other chronic respiratory diseases originating in the perinatal period.

**Congenital heart diseases:** CCS group 213 (This includes ICD-10 codes Q20, Q21, Q22, Q23, Q24, Q25, Q26, Q27, Q28.)

**Nervous system congenital anomalies:** CCS group 216 (This includes ICD-10 codes Q00 to Q07 which incorporates conditions such as spina bifida, anencephaly and other congenital malformations of the nervous system.)

**Other congenital anomalies & perinatal conditions:** CCS groups 224 and 217 (This includes a broad range of congenital anomalies and perinatal conditions with ICD-10 P- and Q- codes, excluding those included within other definitions listed above, such as codes for chronic lung disease.)

**Down’s syndrome:** ICD-10 code Q90

**Cerebral palsy:** ICD-10 code G80

# 
