# Supplementary material for: Risk Factors for Hospital Admission with RSV Bronchiolitis in England: A Population-Based Birth Cohort Study
Source: PLoS One. 2014 Feb 26;9(2):e89186. doi: 10.1371/journal.pone.0089186 (PMC3935842; doi:10.1371/journal.pone.0089186)
Supplement: Appendix S2 — Table S1: Characteristics of birth cohort and infants hospitalised with bronchiolitis. (DOCX) [file pone.0089186.s002.docx]

# Appendix S2

**Table S1: Characteristics of birth cohort and infants hospitalised with bronchiolitis.**

|  | **Birth cohort**  **N** | **%** | **All bronchiolitis**  **n (%)** | **RSV bronchiolitis**  **n (%)** | **Unspecified bronchiolitis**  **n (%)** |
| --- | --- | --- | --- | --- | --- |
| **Number of births** | 296618 | 100.0 | 7189 (2.4) | 2015 (0.7) | 5174 (1.7) |
| **Sex** |  |  |  |  |  |
| Male | 151897 | 51.2 | 4257 (2.8) | 1156 (0.8) | 3101 (2.0) |
| Female | 144659 | 48.8 | 2927 (2.0) | 859 (0.6) | 2068 (1.4) |
| Unknown | 62 | 0.0 | 5 (8.1) | 0 (0.0) | 5 (8.1) |
| **Place of birth** |  |  |  |  |  |
| NHS hospital | 290057 | 97.8 | 6942 (2.4) | 1968 (0.7) | 4974 (1.7) |
| Home | 5728 | 1.9 | 212 (3.7) | 35 (0.6) | 177 (3.1) |
| Unknown | 833 | 0.3 | 35 (4.2) | 12 (1.4) | 23 (2.8) |
| **Birth weight (grams)** |  |  |  |  |  |
| < 1500 | 5442 | 1.8 | 262 (4.8) | 60 (1.1) | 202 (3.7) |
| ≥1500 < 2000 | 2357 | 0.8 | 159 (6.7) | 57 (2.4) | 102 (4.3) |
| ≥2000 < 2500 | 9748 | 3.3 | 407 (4.2) | 118 (1.2) | 289 (3.0) |
| ≥2500 < 3000 | 40632 | 13.7 | 1164 (2.9) | 334 (0.8) | 830 (2.0) |
| ≥3000 < 3500 | 86654 | 29.2 | 2003 (2.3) | 542 (0.6) | 1461 (1.7) |
| 3500+ | 96090 | 32.4 | 2070 (2.2) | 572 (0.6) | 1498 (1.6) |
| Unknown | 55695 | 18.8 | 1124 (2.0) | 332 (0.6) | 792 (1.4) |
| **Gestational age (weeks)** |  |  |  |  |  |
| <37 | 22215 | 7.5 | 1050 (4.7) | 328 (1.5) | 722 (3.3) |
| ≥ 37 ≤ 41 | 212282 | 71.6 | 4637 (2.2) | 1352 (0.6) | 3285 (1.5) |
| ≥ 42 | 10587 | 3.6 | 189 (1.8) | 56 (0.5) | 133 (1.3) |
| Unknown | 51534 | 17.4 | 1313 (2.5) | 279 (0.5) | 1034 (2.0) |
| **Multiple births** |  |  |  |  |  |
|  | 2891 | 1.0 | 94 (3.3) | 30 (1.0) | 64 (2.2) |
| **Specialist neonatal care admission at birth recorded** | | | | | |
|  | 18284 | 6.2 | 845 (4.6) | 280 (1.5) | 565 (3.1) |
| **Strategic Health Authority (SHA) of birth** |  |  |  |  |  |
| North East | 19076 | 6.4 | 636 (3.3) | 248 (1.3) | 388 (2.0) |
| North West | 57424 | 19.4 | 1767 (3.1) | 438 (0.8) | 1329 (2.3) |
| Yorkshire & the Humber | 27897 | 9.4 | 927 (3.3) | 257 (0.9) | 670 (2.4) |
| East Midlands | 24269 | 8.2 | 541 (2.2) | 131 (0.5) | 410 (1.7) |
| West Midlands | 26001 | 8.8 | 587 (2.3) | 139 (0.5) | 448 (1.7) |
| East of England | 37933 | 12.8 | 790 (2.1) | 276 (0.7) | 514 (1.4) |
| London | 65493 | 22.1 | 1025 (1.6) | 327 (0.5) | 698 (1.1) |
| South East Coast | 14917 | 5.0 | 325 (2.2) | 81 (0.5) | 244 (1.6) |
| South Central | 17268 | 5.8 | 454 (2.6) | 61 (0.4) | 393 (2.3) |
| South West | 6340 | 2.1 | 137 (2.2) | 57 (0.9) | 80 (1.3) |
| **Month of Birth** |  |  |  |  |  |
| January | 25579 | 8.6 | 366 (1.4) | 89 (0.3) | 277 (1.1) |
| February | 23824 | 8.0 | 327 (1.4) | 73 (0.3) | 254 (1.1) |
| March | 23481 | 7.9 | 330 (1.4) | 69 (0.3) | 261 (1.1) |
| April | 23192 | 7.8 | 386 (1.7) | 97 (0.4) | 289 (1.2) |
| May | 25276 | 8.5 | 478 (1.9) | 125 (0.5) | 353 (1.4) |
| June | 24132 | 8.1 | 526 (2.2) | 115 (0.5) | 411 (1.7) |
| July | 25254 | 8.5 | 701 (2.8) | 189 (0.8) | 512 (2.0) |
| August | 25907 | 8.7 | 807 (3.1) | 230 (0.9) | 577 (2.2) |
| September | 25724 | 8.7 | 1000 (3.9) | 277 (1.1) | 723 (2.8) |
| October | 25513 | 8.6 | 974 (3.8) | 331 (1.3) | 643 (2.5) |
| November | 24086 | 8.1 | 770 (3.2) | 265 (1.1) | 505 (2.1) |
| December | 24650 | 8.3 | 524 (2.1) | 155 (0.6) | 369 (1.5) |
